# Access to Functional Handwashing Facilities and Associated Factors among South Sudanese Refugees in Rhino Camp Settlement, Northwestern Uganda

**DOI:** 10.1155/2020/3089063

**Published:** 2020-03-30

**Authors:** Frank Namara, Hilbert Mendoza, Gloria Tumukunde, Solomon Tsebeni Wafula

**Affiliations:** ^1^Department of Disaster Risk Management, Uganda Red Cross Society, Kampala, Uganda; ^2^Faculty of Health Science, Uganda Martyrs University, Kampala, Uganda; ^3^Department of Disease Control and Environmental Health, School of Public Health, College of Health Sciences, Makerere University, Kampala, Uganda

## Abstract

**Background:**

Hand hygiene in refugee camp settlements remains an important measure against diarrhoeal infections. Refugee settings are characterised by overcrowding and inadequate access to water and hygiene facilities which favour proliferation of faecal-oral diseases. Handwashing with soap and water is therefore an effective way of preventing such diseases. Despite this knowledge, there is limited information about access to functional handwashing facilities in these settings and associated factors in Uganda.

**Methods:**

Quantitative data were collected from 312 refugee households in Rhino Camp Settlement, Northwestern Uganda, using a semistructured interviewer-administered questionnaire. A modified Poisson regression was used to obtain prevalence ratios (PRs) and 95% confidence intervals (CIs) for the determinants of access to a functional handwashing facility among refugee households. All analyses were performed using STATA 14.0 statistical software.

**Results:**

Of the 312 households, 123 (39.4%) had access to a handwashing facility, but only 72 (23.1%) of households had handwashing facilities that were functional. Duration of stay in the camp exceeding 3 years (adjusted PR = 2.63; 95% CI (1.73–4.00)) and history of receiving home-based education on hand hygiene (adjusted PR = 9.44; 95% CI (1.40–63.86)) were independent predictors of access to a functional handwashing facility.

**Conclusion:**

Access to functional handwashing facilities among the refugee households was low. Our findings highlight the need for more and continued handwashing promotional programs, most especially among newly arrived refugees in the camp.

## 1. Introduction

Hand hygiene in emergency settings remains an important measure against the risk of spread of communicable diseases. Evidence from systematic reviews and clinical trials indicate that handwashing with soap can reduce risk of diarrhoeal infections by 30% to 50% [1–3] and risk of acute respiratory infections by 16% to 23% [4]. While there are proven benefits in maintaining a proper hand hygiene behaviour, hand hygiene is not widely observed for instance, the coverage of basic handwashing facilities with soap and water is only 25% globally, while in sub-Saharan Africa, coverage is only 15% [5, 6]. Moreover, it is estimated that about 74% of potential faecal contacts are not followed by handwashing with soap [6].

In refugee settings, the situation is likely to be worse since there is substantial disruption of habitual and cultural norms thereby potentially altering refugees' practices such as handwashing [7]. In such circumstances, faecal-oral diseases tend to proliferate rapidly due to overcrowding and limited access to adequate water and hygiene facilities [8]. Among the common faecal-oral diseases of concern are diarrhoea and viral hepatitis A and E. Unlike in other settings, the mortality burden contributed by diarrhoeal diseases in refugee settlements could be as high as 40% [9].

In Rhino camp settlement, Uganda, aid agencies such as the International Federation of Red Cross, Uganda Red Cross Society, and World Vision have promoted water, sanitation, and hygiene by ensuring universal access to improved water sources. However, this water supply is often insufficient. Consequently, the limited water tends to be prioritized for drinking and cooking, while hygiene gets short shrift, especially among people who still struggle to understand the connection between hygiene, sanitation, and health [10]. Moreover, the aid agencies carry out health promotion activities such as educating refugees the value of handwashing and even constructing some handwashing facilities. It is, however, the refugees' own decision to change their behaviours.

Currently, it is not known what proportion of households actually have access to these handwashing facilities at their premises. The functionality of these facilities in terms of availability of sufficient water and soap is not known yet. This information could act as a proxy for handwashing behaviour. In our study, we assessed access to functional handwashing facilities and associated factors among refugee households in Rhino settlement camp in Northwestern Uganda. The identification of the determinants of access to handwashing facilities can guide the design of handwashing promotional programs. This is expected to reduce disease burden, health care expenditure at individual, organisational and societal level and consequently improve health and increase productivity among refugees.

## 2. Methods

### 2.1. Study Design and Site

This was a cross-sectional, descriptive study among refugee households in Rhino camp settlement. Rhino camp settlement lies in the Northwestern corner of Uganda, 520 kilometres (km) from Kampala city and only 80 km from the South Sudan border. The camp has existed since 1980s, but in August 2017, the settlement was expanded due to influx of refugees following the South Sudan Civil War and currently hosts over 116,000 refugees. The expansion resulted in establishment of Omugo zone extension area [11]. The camp is administratively divided into seven zones and then further subdivided into thirty nine villages covering an area of 85.525 km^2^ [12]. The map showing the location of Rhino camp settlement can be accessed at https://data2.unhcr.org/en/documents/download/64539. These refugees are mainly from South Sudan and are of diverse ethnic backgrounds, namely, Dinkas, Kuku, Nuer, Kakwa, Madi, and Siluk. The study units for this study were households within the refugee settlement, while adults aged 18 years and above residing in the households within the camp formed the study population.

### 2.2. Sampling Procedures

A total of 14 clusters from the 7 administrative zones (2 clusters per zone) were selected by simple random sampling. For this study, a cluster was taken to be a village which is the lowest local administrative unit in Uganda. Selection of clusters was done by entering all the names of the villages for each zone into a computer and with the help of the random function in Microsoft Excel.

From each selected cluster, a total of 23 households were selected using a systematic random sampling technique leading to an estimation of 322 households. The sampling interval *K* for each village was calculated by dividing the total number of households in the village by the number of households targeted for inclusion in the study (23). The local leader provided the information on household population of his/her village.

A compass was used to identify the starting direction. The research team, guided by the village leader, located the centre of the village and then used a compass to identify the northern direction as the starting direction for household interviews. Every *K*^th^ household from the centre of the village in the Northern direction was considered until the village boundary was reached and the same pattern was repeated in different directions clockwise.

The research assistants then interviewed only one participant per sampled household. In cases where there was more than one eligible participant, simple random sampling was used to select a respondent to answer the questionnaire. This was done by randomly picking from a bag a folded ballot paper with a number corresponding to the participant's name who was then interviewed.

### 2.3. Data Collection

Data were collected in August 2018 using an interviewer-administered semistructured questionnaire. The original English questionnaire was translated to Arabic, the local language spoken by the refugees. Responses were recorded on the English questionnaire. Before using the tool, the Arabic tool was back translated to English to check for consistency. Data were collected on sociodemographic characteristics, water sources, hand hygiene facilities, and knowledge related to hand hygiene and health. The questionnaire was developed based on reviewed literature on handwashing in refugee settings [7, 13, 14]. The data collection tool was pretested in Bidibidi refugee camp, Northwestern Uganda, which has many similarities with the study area. Research assistants were trained on appropriate methods of data collection. Data were collected from all households where participants consented to participate in the study.

### 2.4. Data Management and Statistical Analysis

Data were entered in Epidata 3.02 (EpiData Association, Denmark) and analysed using STATA 14.0 statistical software (StataCorp, Texas, USA). Descriptive analyses such as frequencies, proportions, and means (*where appropriate*) were performed for respondent characteristics (marital status, education level, religion, and age), accessibility to improved water sources, health education sessions on hand hygiene, and access to handwashing facilities. The outcome variable (household access to a functional handwashing facility) was dichotomized and was derived as follows. A household was considered to have a functional handwashing facility if (1) there was a handwashing facility or specific place set for handwashing and (2) cleansing materials (i.e., soap and ash) and water were available at that facility/specific place. To assess the association between access to a functional handwashing facility and each explanatory variable, we applied a generalized linear model of the Poisson family, with logarithm as the canonical link function and applying robust error variance to obtain the crude prevalence ratios (PRs) and their corresponding 95% confidence intervals (CIs). Variables that had threshold probability *p* value ≤0.2 in bivariable models were all added into the multivariable regression model and a stepwise backward elimination method was used until only significant predictors were retained in the model. Crude PRs and adjusted PRs were reported. All *p* values were two-sided and considered significant if less than 0.05.

### 2.5. Ethical Considerations

Ethical approval to conduct the study was obtained from Uganda Martyrs University. The approval to conduct the study within Rhino camp settlement was also sought from the Office of Prime Minister and the authorities at Rhino camp settlement. Participation in the study was voluntary and participants provided written informed consent before the interviews. Identification numbers instead of names of the respondents were used during the research and the data collected were treated with utmost confidentiality.

## 3. Results

### 3.1. Sociodemographic Characteristics of the Households

A total of 312 people living in the refugee camp were recruited into the study representing a 97% response rate. The age of participants ranged from 18 to 75 and had a mean age of 32.7 years (standard deviation/SD ± 10.9). Majority of the respondents were females 70.8% (221/312) and had lived in the camp for less than 3 years 74.0% (231/312). Three quarters 67.9% (212/312) were married and nearly half 47.1% (147/312) had no formal education (Table 1).

### 3.2. Access to Improved Water Sources and Handwashing Practices

In Rhino camp settlement, all households had access to improved water sources. The most popular of these water sources were public tap stands 205 (65.7%) followed by boreholes 104 (33.3%). Overall, a total of 123 households (39.4%) had access to a handwashing facility at theirpremises. Only 72 households (23.1%) had handwashing facility with both soap/ash and water. These households, therefore, were considered as having access to a functional handwashing facility (Table 2).

### 3.3. Independent Predictors of Access to Functional Handwashing Facilities

Bivariate regression analysis indicated that having adequate knowledge compared to limited knowledge that handwashing prevents diarrhoeal infections, receiving home-based education on hand hygiene within the last 6 months, and longer duration of stay in the camp were associated with access to a functional handwashing facility. After adjusting for covariates in the multivariable regression model, households that had stayed in the camp for more than 3 years were 2.6 times more likely to have access to a functional handwashing facility compared to those who had stayed for less than 3 years (adjusted PR = 2.63, 95% CI (1.73–4.00), and *p* value <0.001). Households that had received home-based education sessions in the last six months had higher likelihood of having a functional handwashing facility compared to those who had not received home-based education session in the same time period (adjusted PR = 9.44, 95% CI (1.40–63.86), and *p* value = 0.021) (Table 3).

## 4. Discussion

This study sought to understand household access to functional handwashing facilities and associated factors in Rhino camp settlement in Arua district, Northwestern Uganda. Our findings revealed that household access to functional handwashing facilities was low. Furthermore, household access to functional handwashing facilities was associated with the duration of stay in the camp and history of receiving home-based education on hand hygiene.

Our study findings showed that approximately 3 in 10 households had access to a handwashing facility supplied with soap and water. In contrast, a study in refugee camps in South Sudan found that handwashing facilities existed in more households (64.3%) and (34.9%) of the handwashing facilities had water and soap [7]. In our study, access to a functional handwashing facility was assessed by direct observation, which is only a surrogate marker indicator and not a direct indicator of handwashing behaviour, and the evidence of how well it predicts behaviour is still forthcoming. It can only be assumed that if soap and water are available, then people may be more cautious about hygienic handwashing practices. We believe that actual handwashing practice could be lower since possession of these handwashing facilities does not always translate into expected practices. Indeed studies conducted in similar refugee settings have reported very low prevalence of appropriate handwashing practices despite possession of functional handwashing stations with soap and water in most households [7, 13]. Poor handwashing practices are sometimes linked to gaps in the health information provided to the community by hygiene promoters and community health workers responsible for hygiene promotion and community mobilisation [15].

Insufficient soap can be a major barrier to handwashing, often resulting into using the same piece of soap for multiple purposes which may include laundry and bathing [16]. In our study, we found that although a large majority of the households used soap regularly, approximately 4 in 10 of those households did not report using soap during handwashing at critical times citing reasons such as high cost of soap and lack of soap in households. This implies that household members have to forego soap during the washing of hands sometimes which is a concern and obviously, in such scenarios, thorough handwashing may not be possible, creating conditions that increase risk of diarrhoeal infections.

In multivariable analysis, length of stay in the camp showed a significant association with household access to a functional handwashing facility. Households that had stayed for at least 3 years in the settlement camp were more likely to have access to a functional handwashing facility than those that stayed for less than 3 years. This is in line with evidence that suggests that people who have lived longer in refugee camps are expected to have more frequent exposure to hygiene promotion activities and can adopt good hygiene practices such as possession of functional handwashing facilities [17]. In contrast, similar studies in other refugee camps had contradictory findings [7, 13, 15]. In these studies, it was hypothesized that when people stay longer in the camps, they tend to become complacent on many hygiene issues as compared to new arrivals. Further research is needed to assess and compare the level of complacency between newly arrived refugees and those that have stayed longer in the camps and how this influences uptake of hygiene interventions.

Our study also indicated that households that received basic home-based education sessions on handwashing within 6 months preceding the survey were more likely to have access to functional handwashing facilities. Health education programs on hygiene have shown preeminence over only investing in handwashing facilities in improving handwashing behaviour, and this is due to the belief that behaviour is learned and consolidated [18]. A study conducted among children in a humanitarian setting revealed that children who had received home-based interactive sessions had a higher likelihood to wash hands after key handwashing sessions [19]. These findings suggest that home-based education sessions have potential to encourage good handwashing practices among households and should therefore be promoted. An evaluation in a Burundian refugee camp stressed that high coverage of community mobilisation and hygiene education may increase knowledge levels and influence positive behavioural changes among refugees in camp settings [15]. Thus, besides having home-based education, the methods of community mobilisation and hygiene education need to be appropriate for different camp settings to ensure sufficient knowledge levels and behavioural changes. Such settings may also require constant repetition and reinforcement of information required for behaviour change. In addition, we stress the need to address the potential barriers to handwashing such as the convenience, availability of water in addition to sensitization, and health education sessions to achieve behavioural change [20].

A limitation of our study is that we assessed access to functional handwashing facility, which though reliable, is only a proxy indicator and does not give a more accurate estimate for handwashing practice. Better estimates can be obtained from long-term direct observation methods on handwashing. However, this study provides useful insights into access to functional handwashing facilities and its associated factors among refugee households in Northwestern Uganda. Areas of further research could include conducting studies on handwashing practices using direct observation methods and for an extended period.

## 5. Conclusion

Access to a functional handwashing facility among refugee households was low. In order to improve access to a functional handwashing facility, there is need to ensure availability of soap at the sites for handwashing and, more critically, home-based health education programs on hand hygiene should be intensified. Longer duration of stay in the camp was also associated with access to a functional handwashing facility. We suggest continuous home-based education programs on hand hygiene, particularly focussing on newly arrived refugees in the camps.

## Figures and Tables

**Table 1:** Sociodemographic characteristics of the respondents.

Sociodemographic characteristics	Category	Number of participants (*N* = 312)	Summary measure
Gender	Female	221	70.8%
	Male	91	29.2%

Age in years	18–36	225	72.1%
	37–55	68	21.8%
	>55	19	6.1%
	Mean (SD)	312	32.7 (10.9)

Ethnic tribe	Dinka	80	25.6%
	Nuer	27	8.7%
	Kuku	23	7.4%
	Kakwa	52	16.7%
	Madi	15	4.8%
	Siluk	5	1.6%
	Others	110	35.3%

Religion	Christians	308	98.7%
	Muslims	04	1.3%

Marital status of respondents	Single	100	32.1%
	Married/cohabiting	212	67.9%

Duration of stay in a camp in years	≤3 years	231	74.0%
	>3 years	81	26.0%
	Mean (SD)	312	7.1 (3.4)

Level of education	No formal education	147	47.1%
	Primary	4	1.3%
	Postprimary	161	51.6%

Household size	≤5 members	96	30.8%
	>5 members	216	69.2%
	Mean (SD)	312	2.9 (2.7)

Others included Muru, Pojulu, and Baka.

**Table 2:** Water sources and access to handwashing facilities.

Variable	Category	Number of participants	Summary measure
Access improved water source	Yes	312	100.0%

Types of water sources	Public tap stands	205	65.7%
	Boreholes	104	33.3%
	Private tap stands	3	1.0%

Handwashing facility present	No	189	60.6%
	Yes	123	39.4%

Type of handwashing facility	Tippy taps	104	84.6%
	Oxfam buckets	9	7.3%
	Handwashing bags	9	7.3%
	Bush proof handwashing containers	1	0.8%

Household uses soap	No	39	12.5%
	Yes	273	87.5%

Soap available for handwashing at critical times	No	120	44.0%
	Yes	153	56.0%

Reasons for unavailability of soap at handwashing stations^*∗*^	High cost of soap	83	69.1%
	Lack of soap in the house	58	48.3%
	Use ash instead of soap	79	65.8%

Handwashing facility has soap and water	No	240	76.9%
	Yes	72	23.1%

Household received home-based health education on hand hygiene in the last 6 months	No	45	14.4%
	Yes	267	85.6%

^*∗*^Multiple options.

**Table 3:** Independent predictors of access to functional handwashing facilities among refugee households.

Characteristic	Access to a functional handwashing facility		Crude PR (95% CI)	*p* value	Adjusted PR (95% CI)	*p* value
	Yes, *n* (%)	No, *n* (%)				
Marital status of household head						
Single	19 (19.0)	81 (81.0)	1			
Married	53 (25.0)	159 (75.0)	1.32 (0.82–2.10)	0.250		

Household size						
≤5 members	18 (18.8)	78 (81.2)	1			
>5 members	54 (25.0)	162 (75.0)	1.33 (0.83–2.15)	0.237		

Household member suffered diarrhoea in the last 30 days						
No	40 (21.9)	143 (78.1)	1			
Yes	32 (24.8)	97 (75.2)	1.13 (0.76–1.71)	0.543		

Duration of stay in camp						
≤3 years	35 (15.2)	196 (84.8)	1		1	
More than 3 years	37 (45.7)	44 (54.3)	3.01 (2.05–4.44)	**<0.001**	2.63 (1.73–4.00)	**<0.001**

Handwashing is good preventive strategy to diarrhoea						
No	14 (15.4)	77 (84.6)	1			
Yes	56 (29.2)	136 (70.8)	1.89 (1.11–3.22)	**0.018**		

Received home-based education session on handwashing in the last 6 months						
No	1 (2.2)	44 (97.8)	1		1	
Yes	71 (26.6)	196 (73.4)	11.96 (1.70–84.22)	**0.013**	9.44 (1.40–63.86)	**0.021**

CI, confidence interval; PR, prevalence ratio.

## Data Availability

The dataset used to support the findings of this study is available from the corresponding author upon request.

## Supplementary Materials

Supplementary Materials Supplementary File 1: interview questionnaire for the study (PDF).
