# Supplementary material for: Access to Functional Handwashing Facilities and Associated Factors among South Sudanese Refugees in Rhino Camp Settlement, Northwestern Uganda
Source: J Environ Public Health. 2020 Mar 30;2020:3089063. doi: 10.1155/2020/3089063 (PMC7149417; doi:10.1155/2020/3089063)
Supplement: Supplementary Materials — Supplementary File 1: interview questionnaire for the study (PDF). [file 3089063.f1.pdf]

Supplementary 1: Questionnaire

HOUSEHOLD INDENTIFICATION PARTICULARS (HI)		
**NO.**	**QUESTIONS**	**RESPONSE**
HI01	Date of interview (day/month/2018)	___/___/2018
HI02	Start time	
HI03	Village	
**HI04**	**Household Sample Number**	
HI05	Name of data collector	

HOUSEHOLD SOCIOECONOMIC AND DEMOGRAPHIC CHARACTERISTICS (HD)			
**NO.**	**QUESTIONS**	**RESPONSE AND CODING CATEGORIES (Circle the code that is given by the respondent)**	**SKIP PATTERNS AND IMPORTANT NOTES**
HD01	Gender	1= Female<br>2= Male	
HD02	How old are you? **(as of last birthday, record age in complete years)**	_____________ years	
HD03	To which tribe do you belong?	1= Dinka<br>2= Nuer<br>3= Kuku<br>4= Kakwa<br>5= Madi<br>6= Siluk<br>7= Other (specify) ______________________________	
HD04	What is your religion?	1= Catholic<br>2= Anglican<br>3= Muslim<br>4= Pentecostal<br>5= Seventh Day Adventists (SDA)<br>6= Other (**specify)** ______________________________	
HD05	What is your marital status?	1= Single<br>2= Married/cohabiting<br>3= Widowed<br>4= Divorced/separated	
HD06	Have you ever attended school?	1= Yes<br>2= No →	**Skip to HD08**
HD07	What is your highest level of education attained?	1= Primary (P1 to P7)<br>2= O level (S1 to S4)<br>3= A level (S5 to S6)	

		4= Tertiary/ University	
HD08	How many people live in your household? (include both children and adults)		
HD09	How long have you lived in Rhino camp?	_____________ (years)<br>_____________ (Months)	
**THE NEXT QUESTIONS ASK ABOUT DIARRHEAL DISEASES (DD)**			
DD01	During the past 30 days has anyone in your family suffered from diarrheal diseases	1= Yes<br>2= No	
DD02	What do you think can cause diarrhea?	1=Bad/ dirty water<br>2= Bad/dirty food<br>3= Poor hygiene<br>4= Feces/ defecating in the open<br>5= Dirty hands<br>6= Germs<br>7= Flies<br>8= Other (specify)<br>____________________________<br>9= Do not know	
DD03	Do you think diarrhea can be prevented?	1= Yes<br>**2= No**<br>3= Don't **know**	**Skip to HWA01**
DD04	What are some of the ways of preventing diarrhea?	1= Wash hands<br>2= Use soap<br>3= Use toilet facility to defecate<br>4= Dispose children's feces in toilet<br>5= Bury feces<br>6= Drink clean water<br>7= Store water safely<br>8= Treat water (boil, filter, chlorinate)<br>9= Prepare food hygienically<br>10= Dispose of rubbish in pit<br>11= Breast feeding<br>12= Measles vaccination<br>13= Good nutrition<br>14= Other (specify)<br>__________________________<br>15= Do not know	
**THE NEXT QUESTIONS ASK ABOUT THE HAND WASHING FACILITIES AT THE HOUSEHOLD LEVEL (HWA)**			
**NO.**	**QUESTIONS**	**RESPONSE AND**	**SKIP PATTERNS**

		**CODING CATEGORIES (Circle the code that corresponds with the response given by the respondent)**	
HWA01	Does the household have a functioning hand washing station (Please observe only)	1= Yes<br>2= No →	**Skip to HWA08**
HWA02	What type of handwashing station is it?	1= Oxfam Buckets<br>2= Tippy taps<br>3= Bush proof hand washing containers<br>4= Hand washing bags<br>5= Other (specify)<br>______________________________	
HWA03	Where is the handwashing facility located?	1= Next to the toilet<br>2= Next to the kitchen<br>3= Next to the house<br>4= Other (specify)<br>______________________________	
HWA04	Is the handwashing facility supplied with water? (Please observe to see if there is water within the water storage container of the hand washing facility)	1= Yes<br>2= No	
HWA05	Is their soap at hand washing facility (Please observe)	1= Yes<br>2= No	
HWA06	Does the hand washing facility include resources for hygienic hand drying?	1= Yes<br>2= No	
HWA07	How many people use the handwashing station	___________ people<br>______________	
HWA08	If there is no hand washing facility within the household. What is the main reason for it being absent?	______________________________<br>______________________________<br>______________________________<br>______________________________<br>______________________________	
HWA09	What is your household main source of water?	1=Own tap<br>2=Borehole<br>3=Stream/river<br>4=Public tap<br>5=Protected well<br>6=Unprotected well<br>7=Other (specify)<br>______________________________	

HWA10	What is the distance to the main source of water (15 minutes= 1km)	1= Within 500m<br>2= 1 to 2km<br>3= Other (Specify)<br>______________________	
HWA11	Do you use any soap in your household?	1= Yes<br>2= No →	**Skip to KHW01**
HWA12	What is your main source of soap?	1= Retail shops<br>2= Donations from organizations<br>3= Other (specify)<br>______________________	
HWA13	What is soap reserved for in your household (high priority usage areas)? Rank in order of priority 1,2,3,4 **(1=very high 2=High 3= Low 4= Very low)**	Laundry…………………<br>Washing dishes……………<br>Bathing………………<br>Handwashing……………	
HWA14	Where does your household prefer keeping the soap?	1= Place on a shelf in the kitchen or on top of a kitchen cupboard<br>2= Keep soap under furniture such as bed<br>3= Keep soap on a dish rack near the toilet<br>4= No specific place for washing hands<br>5= Far from main house<br>6= At communal handwashing facility<br>7= At a place where children cannot reach<br>8= Other (specify)<br>______________________	
HWA15	Is soap always available for hand washing at critical times of handwashing?	1= Yes →<br>2= No	**Skip to KHW01**
HWA16	What are the main reasons for not using soap for handwashing at the critical times?	1= Cost of soap (cannot afford)<br>2= Not available in area shops<br>3= No soap is available in the house<br>4= Other (specify)<br>______________________	
HWA17	In case soap is not available, what do you use for hand washing?	1= Ash<br>2= Mud<br>3= Nothing<br>4= Other (specify)	

		___	
HWA18	What would it take you to make handwashing with soap at critical times a habit?	1= Ease access of water<br>2= Able to afford soap<br>3= Ease access to ash and water<br>4= Other (specify)<br>___	
**THE NEXT QUESTIONS ASK ABOUT KNOWLEDGE ABOUT HAND WASHING WITH SOAP (KHW)**			
KHW01	When are the critical times when handwashing with soap should be done?	1= After visiting the toilet<br>2= Before handling food<br>3= After handling food<br>4= After cleaning babies bottom<br>5= Other (specify)<br>___<br>6= Do not know	
KHW02	What is the primary purpose of hand hygiene?	1= To reduce germs on hands<br>2= To keep hands clean<br>3= To keep nails clean<br>4= Do not know<br>5= To keep the hands smooth<br>6= To avoid diseases<br>7= Other<br>___	
KHW03	Does Human feaces contain germs?	1= Yes<br>2= No<br>3= Do not know	
KHW04	Does human urine contain germs?	1= Yes<br>2= No<br>3= Do not know	
KHW05	Does poor hand washing cause disease?	1= Yes<br>2= No<br>3= Do not know	
KHW06	Is water only enough for hand washing?	1= Yes<br>2= No<br>3= Do not know	
KHW07	Is it necessary to wash hands with soap?	1= Yes<br>2= No<br>3= Do not know	
KHW08	If, yes, why would you use soap	1= Personal care<br>2= Attraction<br>3= Health (freedom from germs and diseases<br>4= Other (specify)<br>___	
**THE NEXT QUESTIONS ASK ABOUT PARTICIPATION IN HOUSEHOLD VISITS AIMED AT PROMOTING HAND WASHING (HHP)**			

HHP01	Have you received a household visit from a hygiene promoter to educate you about hand washing in the last 6 months?	1= Yes<br>2= No →	**End Interview**
HHP02	Which hand washing information is mostly disseminated during the household visits?	1=Why you should wash hands<br>2=Why you should use soap to wash hands<br>3=Why you should wash hands with clean water<br>4=When you should wash hands<br>5=What to do if you witness a person not washing hands after a critical time<br>6=Location to place soap<br>7=Other ______________________	
HHP03	Did they demonstrate to you how to wash your hands properly?	1= Yes<br>2= No	
HHP04	Did they demonstrate to how to build your hand washing station?	1= Yes<br>2= No	
HHP05	Did they come with any materials like educational posters, charts about handwashing in any of their visits?	1= Yes<br>2= No	
HHP06	In your opinion how would you rate the household visits by the hygiene promoters?	1= Excellent<br>2= Good<br>3= Fair<br>4= Bad/poor<br>5= Very poor<br>6= Do not know	
